# Organizational Factors Influencing Progress in Taiwan’s Disability Prevention Programs

**DOI:** 10.1093/geroni/igaf122.3988

**Published:** 2025-12-31

**Authors:** Wan-Yu Chiu, Hui-Fen Mao, Ling-Hui Chang, Ya-Mei Chen

**Affiliations:** National Taiwan University, Zhongzheng District, Taipei, Taiwan (Republic of China); National Taiwan University, Taipei City, Taipei, Taiwan (Republic of China); National Cheng Kung University, 701, Tainan, Taiwan (Republic of China); National Taiwan University, National Taiwan University, Taipei, Taiwan (Republic of China)

## Abstract

Taiwan’s nationwide Prevention and Delay of Disability (PDD) program plays a key role in promoting older adults’ function. However, health progress varies by frailty level and may be shaped by organizational characteristics. This study investigated how such characteristics influence different types of health progress among older adults. The cross-sectional study (2019–2020) involved 392 administrators from PDD service centers and 13,213 participants, categorized as frail (563), pre-frail (2,397), or healthy (10,253). Over a 12-week period, multiple centers implemented a multi-domain intervention composed of similar elements, such as physical exercise, nutrition, cognitive training, and social engagement. Organizational-level data were collected from administrators using six dimensions: efficiency, responsiveness, professionalism, cooperation, environment, and sustainability. Health progress among participants was classified into three distinct types for each frailty level: stable, multidimensional, and unique, resulting in a total of 9 progress types. Using multilevel analysis, higher organizational responsiveness was associated with greater progress among frail and pre-frail participants. Frail individuals were more likely to exhibit depressive-social progress (β = 0.746, p = 0.013), while pre-frail participants showed a tendency toward multidimensional progress (β = 0.312, p = 0.012). Organizations managing a greater variety and volume of programs were more likely to facilitate multidimensional progress in both frail (β = 0.148, p = 0.004) and pre-frail groups (β = 0.034, p = 0.008), while organizational factors appeared to have limited influence on healthy participants. These findings highlight the role of organizational factors in influencing health outcomes among older adults.

